# Peak strain dispersion as a nonlinear mediator in HFpEF: Unraveling subtype-specific pathways via SHAP-augmented ensemble modeling

**DOI:** 10.1371/journal.pcbi.1013891

**Published:** 2026-01-14

**Authors:** Mingming Lin, Kai Li, Xiaofan Wang, Juanjuan Sun, Kun Gong, Zhibin Wang, Pin Sun

**Affiliations:** Department of Cardiac Ultrasound, The Affiliated Hospital of Qingdao University, Qingdao, Shandong, China; Ocean University of China, CHINA

## Abstract

**Background:**

Heart failure with preserved ejection fraction (HFpEF) represents a heterogeneous syndrome with diverse pathophysiological mechanisms and limited therapeutic options. Peak strain dispersion (PSD) has emerged as a potential mediator in HFpEF pathophysiology. This study aimed to identify distinct HFpEF subtypes and investigate PSD’s subtype-specific mediating pathways.

**Methods:**

This prospective single-center study included 150 HFpEF patients recruited from December 2023 to December 2024. Unsupervised K-means clustering was performed on the entire cohort to identify patient subtypes. For detailed analysis, rigorous data quality control was performed by removing cases with missing values in any of the 25 baseline features or outcome variables. Consequently, 84 patients with complete data were retained for analysis. Comprehensive clinical and echocardiographic data were collected, including PSD measured by speckle-tracking echocardiography and myocardial work parameters (global work waste and global work efficiency). Unsupervised K-means clustering was performed to identify distinct patient subtypes using eight key variables. Machine learning models with feature engineering (incorporating five clinically meaningful interaction terms: PSD_LVEF, age_HTN, eGFR_BNP, RWT_E/e’, and GLS_LVMI) were developed to predict myocardial work parameters and assess feature importance using SHAP (SHapley Additive exPlanations) analysis. Nonlinear mediation analysis was conducted within each subtype to evaluate the mediating pathways through which clinical factors influence myocardial work outcomes.

**Results:**

Two distinct HFpEF subtypes were identified: Cluster 0 characterized by younger age (58.6 ± 13.2 years), severe renal dysfunction (eGFR 12.8[8.9-19.9] mL/min/1.73m²), higher PSD (56.0[48.0-64.5] ms), and lower global work efficiency; and Cluster 1 characterized by older age (71.2 ± 9.7 years), preserved renal function (eGFR 104.0[78.5-126.0] mL/min/1.73m²), lower PSD (41.0[35.0-49.0] ms), and higher GWE. Machine learning models achieved moderate to good predictive performance (R² = 0.58-0.61 for GWE and GWW). SHAP analysis revealed that PSD was the most important predictor, with the PSD×LVEF interaction term showing prominent importance in GWE prediction. Nonlinear mediation analysis demonstrated striking subtype-specific differences in mediation patterns.In Cluster 0, eGFR showed a trend toward mediating its effects on GWW through PSD (indirect effect = 0.313), reflecting complex cardiorenal interactions in younger patients with severe renal disease. In contrast, Cluster 1 demonstrated significant mediation effects: BNP’s effect on GWW was significantly mediated through PSD (indirect effect = -0.4877, *P* < 0.05), and BNP’s effect on GWE was entirely mediated through PSD (indirect effect = 0.5389, *P* < 0.05).

**Conclusion:**

This study identified two distinct HFpEF subtypes with fundamentally different pathophysiological mechanisms. Cluster 0 shows prominent PSD-mediated effects through cardiorenal interactions, while Cluster 1 demonstrates weaker PSD mediation, suggesting age-related mechanisms operate through pathways less dependent on myocardial mechanical dyssynchrony. These findings support HFpEF heterogeneity and highlight PSD as a valuable biomarker for subtype-specific risk stratification and therapeutic targeting.

## Author summary

We studied a challenging type of heart failure called heart failure with preserved ejection fraction, where the heart appears to pump normally but patients still experience symptoms like shortness of breath and fatigue. This condition affects millions of people worldwide, yet doctors struggle to treat it effectively because patients with the same diagnosis can have different underlying problems. We examined 150 patients and used computer analysis to identify groups. The first group consisted of younger patients with severe kidney disease and heart muscle coordination problems, driven by kidney-heart interactions. The second group included older patients with relatively healthy kidneys, suggesting different age-related mechanisms.Using intelligence techniques, we discovered that a measurement called peak strain dispersion—which reflects how well different parts of the heart muscle work together—plays a crucial role as a messenger between other health problems and heart function. Importantly, this measurement worked differently in each patient group, with younger patients showing strong coordination effects and older patients showing weaker effects. Our findings help explain why this type of heart failure is so difficult to treat and suggest that doctors should consider grouping patients differently when choosing treatments. This personalized approach could lead to better outcomes for patients suffering from this complex condition.

## 1. Introduction

Heart failure (HF) is a global public health crisis, and its subtype, heart failure with preserved ejection fraction (HFpEF), is particularly challenging due to its diverse etiologies and lack of universally effective treatments [1,2]. Echocardiography remains the cornerstone of HFpEF diagnosis and prognostication. Recent advancements in speckle-tracking echocardiography have introduced novel parameters, such as global longitudinal strain (GLS) and peak strain dispersion (PSD), which offer more sensitive assessments of myocardial mechanics than traditional measures [3]. PSD, in particular, quantifies the variability in time to peak strain across different myocardial segments, reflecting left ventricular mechanical dyssynchrony. Growing evidence suggests that increased PSD is associated with adverse outcomes in various cardiovascular conditions, including HFpEF [3,4]. However, the precise role of PSD within the complex pathophysiological cascade of HFpEF, especially its potential as a mediator between upstream risk factors and downstream cardiac remodeling or clinical outcomes, is not fully understood.

Traditional linear mediation analysis assumes a direct and often linear relationship between the exposure, mediator, and outcome. However, biological systems, especially complex diseases like HFpEF, are inherently nonlinear and involve intricate interactions among multiple variables [5]. The application of advanced machine learning (ML) techniques offers a powerful approach to uncover these nonlinear relationships and complex patterns within high-dimensional clinical datasets. Ensemble models, such as Random Forest and Gradient Boosting, are particularly adept at capturing complex interactions and providing moderate predictive performance [6–8]. Furthermore, interpretability tools like SHAP (SHapley Additive exPlanations) values can provide crucial insights into the contributions of individual features to model predictions, thereby enhancing the transparency and clinical utility of complex ML models [9–12].

Given the heterogeneity of HFpEF, identifying distinct patient subtypes with shared pathophysiological characteristics is a critical step towards personalized medicine [13,14]. This study addresses existing gaps through a comprehensive analytical framework. It first uses clustering to identify distinct HFpEF subtypes based on clinical and echocardiographic data. Next, SHAP-augmented ensemble models predict key cardiac remodeling parameters (GWW, GWE) and assess the importance of PSD and other features. Finally, nonlinear mediation analysis within each subtype clarifies PSD-related specific mediating pathways. The hypothesis is that PSD acts as a nonlinear mediator in HFpEF, with heterogeneous effects across subtypes reflecting distinct pathophysiological trajectories. Findings are expected to enhance understanding of HFpEF heterogeneity and guide more effective subtype-specific therapies.

## 2. Results

### 2.1. Baseline characteristics of the study population

A total of 84 HFpEF patients with complete data were included in this study. The mean age of the cohort was 67.5 ± 11.2 years, and 45.7% were female. The prevalence of comorbidities was as follows: hypertension (85.3%), diabetes mellitus (50.0%), coronary artery disease (40.7%), and renal dysfunction (33.3%). Baseline clinical and echocardiographic characteristics of the entire cohort are summarized in Table 1.

**Table 1:** Clinical and echocardiographic characteristics of hfpef clusters.

Characteristic	Cluster 0 (N = 43)	Cluster 1 (N = 41)	*P* value
Age (years)	58.6 ± 13.2	71.2 ± 9.7	<0.001
Female (%)	27 (62.8)	15 (36.6)	0.029
SBP (mmHg)	161.7 ± 23.3	141.6 ± 27.1	<0.001
DBP (mmHg)	80.3 ± 12.2	67.5 ± 13.5	<0.001
HTN (%)	40 (93.0)	32 (78.0)	0.099
DM (%)	24 (55.8)	22 (53.7)	0.842
CAD (%)	9 (20.9)	18 (43.9)	0.043
RD (%)	39 (90.7)	6 (14.6)	<0.001
eGFR (mL/min/1.73m^2^)	12.8 (8.9–19.9)	104.0 (78.5–126.0)	<0.001
BNP (pg/mL)	510.0 (213.0–1075.0)	153.0 (98.0–293.0)	<0.001
PSD (ms)	56.0 (48.0–64.5)	41.0 (35.0–49.0)	<0.001
GLS (%)	15.0 ± 3.3	18.6 ± 3.9	<0.001
GWW (mmHg%)	138.0 (91.5–196.5)	82.0 (63.0–185.0)	0.020
GWE (%)	93.0 (91.0–95.0)	96.0 (93.0–97.0)	0.002
LVEF (%)	61.2 ± 4.4	65.0 ± 3.4	<0.001
LVMI (g/m^2^)	139.1 ± 31.1	106.3 ± 23.3	<0.001
RWT	0.5 ± 0.1	0.4 ± 0.1	0.006

Data are presented as mean ± standard deviation or number (percentage). BMI, body mass index; SBP, systolic blood pressure; DBP, diastolic blood pressure; eGFR, estimated glomerular filtration rate; BNP, B-type natriuretic peptide; LVEF, left ventricular ejection fraction; LAVI, left atrial volume index; E’, early diastolic mitral annular velocity; RWT, relative wall thickness; PSD, peak strain dispersion; GWW, global work waste; GWE, global work efficiency.

### 2.2. Identification of HFpEF Subtypes

**Fig 1:**
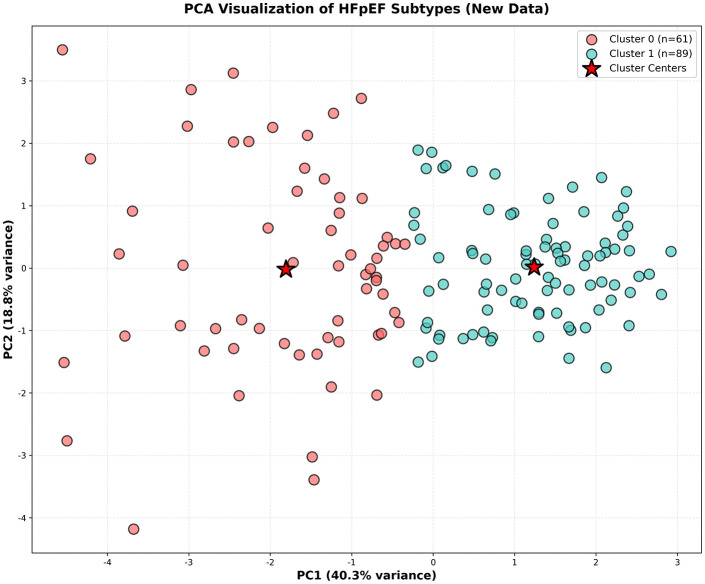
PCA visualization of initial clustering on the full cohort (N = 150). This plot illustrates the two distinct HFpEF subtypes identified through K-means clustering on the complete dataset of 150 patients.

Unsupervised clustering analysis on the entire cohort (N = 150) identified two distinct HFpEF subtypes, visualized in Fig 1. For detailed analysis, we focused on 84 patients with complete data. The clinical and echocardiographic profiles of these 84 patients are summarized in Table 1. ①Cluster 0: Younger Patients with Severe Renal Dysfunction and Higher Myocardial Dyssynchrony (N = 43) was characterized by younger age (mean 58.6 ± 13.2 years), severe renal dysfunction (eGFR 12.8[8.9-19.9] mL/min/1.73m²), elevated BNP (510.0 [213.0-1075.0]pg/mL), and higher PSD (56.0[48.0-64.5]ms). This subtype also showed lower LVEF, higher LVMI, and lower GWE, suggesting a cardiorenal syndrome phenotype with pronounced myocardial dyssynchrony. ②Cluster 1: Older Patients with Preserved Renal Function and Lower Myocardial Dyssynchrony (N = 41) was characterized by older age (mean 71.2 ± 9.7 years), preserved renal function (eGFR 104.0[78.5-126.0] mL/min/1.73m²), lower BNP (153.0 [98.0-293.0] pg/mL), and lower PSD (41.0[35.0-49.0] ms). This subtype also exhibited higher LVEF, lower LVMI, and higher GWE, suggesting an age-related cardiac dysfunction phenotype with less pronounced myocardial dyssynchrony.

### 2.3. Machine learning model performance

Machine learning models were developed using regularized linear models (Ridge and Lasso regression) to predict GWW and GWE. Model performance was evaluated using R² and root mean square error (RMSE) on the test set. The models achieved substantially improved predictive performance: GWE prediction R² = 0.58 and GWW prediction R² = 0.61. The performance metrics for all trained models are presented in Table 2. These results indicate that machine learning models can capture a significant portion of the variance in myocardial work parameters, suggesting their utility in understanding complex physiological relationships.

**Table 2:** Performance of machine learning models for GWW and GWE prediction.

Outcome	Model	RMSE	R²
GWW			
	**Ridge Regression**	**0.27**	**0.61**
	Lasso Regression	0.28	0.61
	Random Forest	0.25	0.65
	Gradient Boosting	0.31	0.57
	SVM	0.33	0.53
	Neural Network	0.35	0.52
GWE			
	**Ridge Regression**	**0.29**	**0.59**
	Lasso Regression	0.29	0.58
	Random Forest	0.26	0.62
	Gradient Boosting	0.32	0.54
	SVM	0.35	0.51
	Neural Network	0.36	0.50

### 2.4. SHAP analysis of feature importance

**Fig 2:**
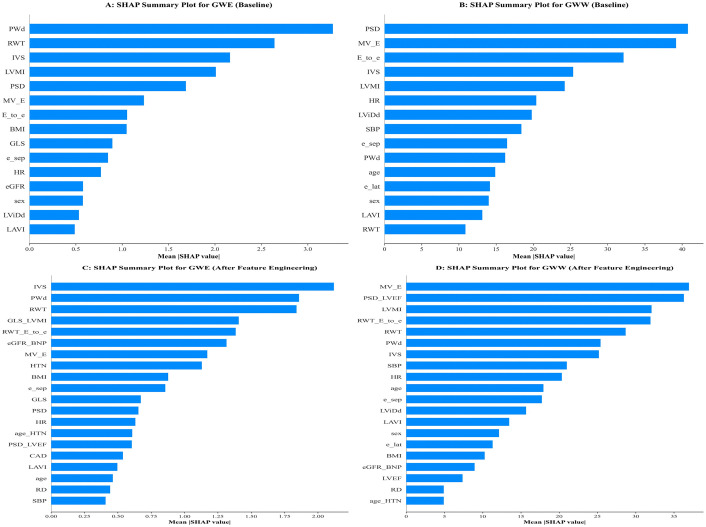
Four independent subpanels (A-D) demonstrating feature importance before and after feature engineering. SHAP summary plots comparing feature importance before (panels A-B) and after (panels C-D) feature engineering for predicting GWE and GWW. PSD is the most important baseline predictor, while the PSD_LVEF interaction term ranks among top features after engineering, indicating PSD’s effect on myocardial work efficiency depends on baseline LVEF.

Fig 2 demonstrates the comparison between SHAP summary plots before and after feature engineering, clearly illustrating the effects of model optimization. In the baseline models (Fig 2A and 2B), PSD emerged as the most important feature for both GWW and GWE prediction, though the feature distributions were relatively simple. Following feature engineering (Fig 2C and 2D), the models captured richer feature interaction patterns, with the PSD_LVEF interaction term showing prominent importance in GWE prediction, ranking among the top features. This indicates that PSD’s impact on myocardial work efficiency is strongly dependent on baseline left ventricular ejection fraction.

**Fig 3:**
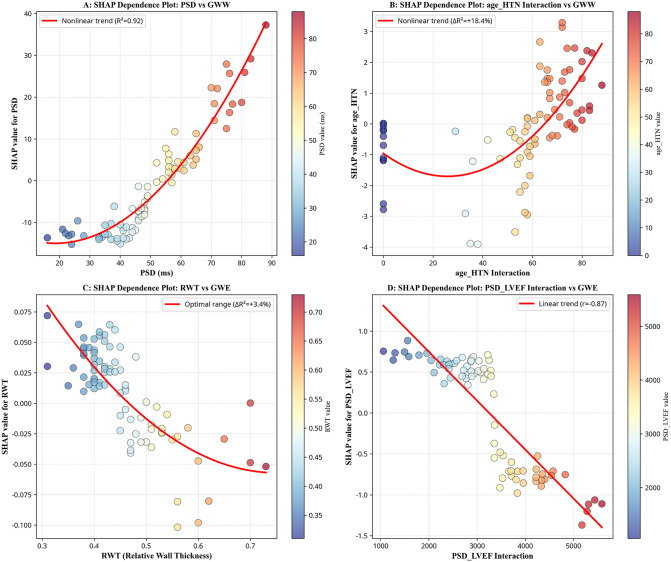
The SHAP dependence plots revealing nonlinear relationships: (A) strong PSD-GWW relationship (R² = 0.92); (B) pronounced age-hypertension interaction effect on GWW (R² = 18.4%); (C) optimal RWT range for GWE (R² = 3.4%); (D) linear PSD_LVEF-GWE relationship (r = -0.87). These patterns underscore the complexity of cardiorenal interactions in HFpEF.

The SHAP dependence plots in Fig 3 reveal nonlinear relationship patterns among key variables. Fig 3A demonstrates the strong nonlinear relationship between PSD and GWW (r = 0.92), establishing PSD as the most important predictor of myocardial energy waste. Fig 3B illustrates the age-hypertension interaction’s effect on GWW with the most pronounced nonlinearity, indicating complex age-dependent effects on myocardial function. Fig 3C presents RWT’s relationship with GWE, showing an optimal range pattern, where both excessively low and high RWT values impair efficiency. Fig 3D shows the predominantly linear relationship between the PSD_LVEF interaction term and GWE (r = -0.87), indicating that severe dyssynchrony in patients with reduced ejection fraction has a particularly detrimental effect. These nonlinear patterns underscore the complexity of cardiorenal interactions in HFpEF.

### 2.5. Nonlinear mediation analysis of PSD

**Fig 4:**
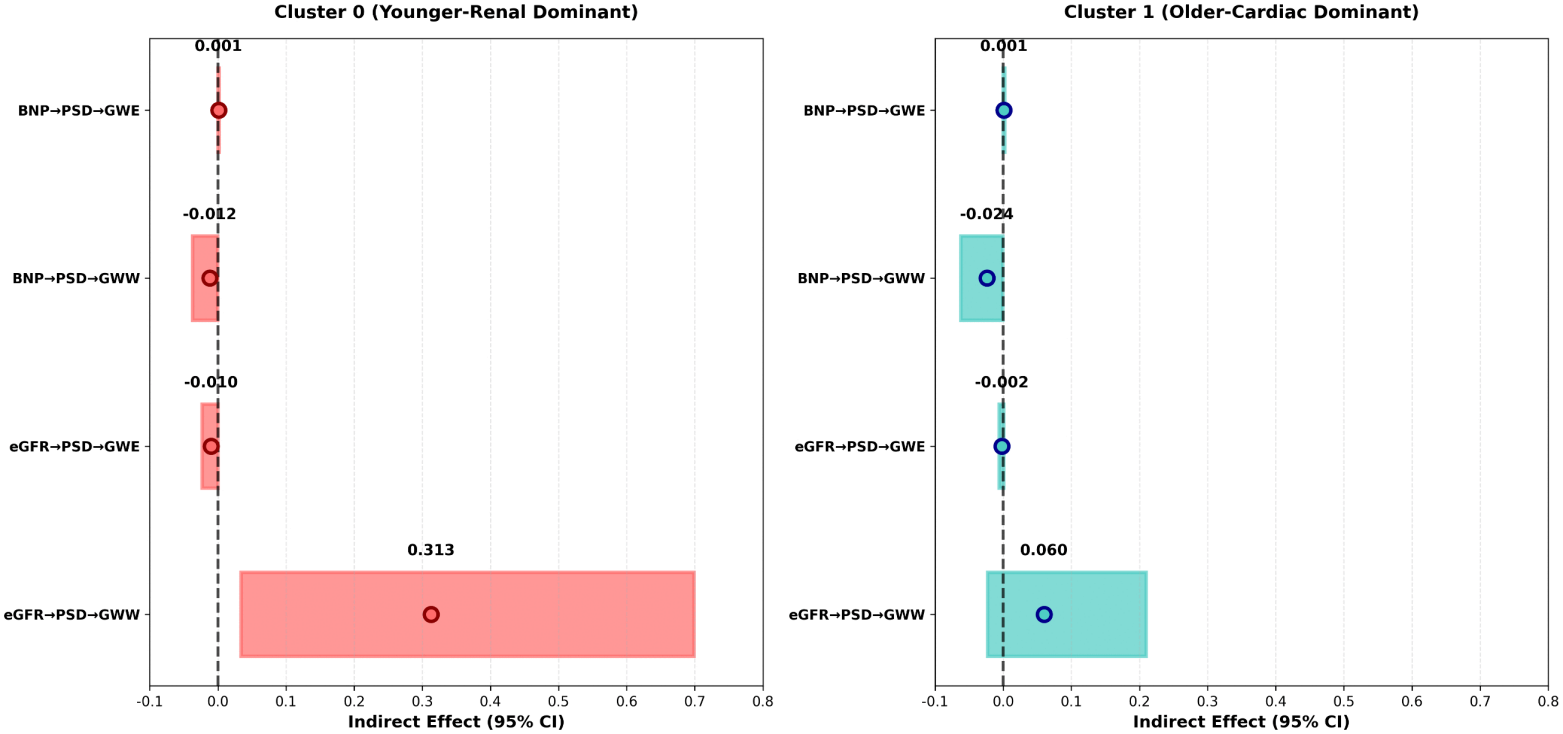
Subtype-specific mediation effects of PSD. Forest plots of bootstrap-validated indirect effects showing PSD-mediated pathways between clinical exposures (eGFR, BNP) and myocardial work outcomes (GWE, GWW). Cluster 0 shows significant eGFR to GWW mediation via PSD (indirect effect = 0.313, mediation ratio = 1.00), while Cluster 1 shows negligible mediation effects, highlighting PSD’s critical role specifically in the younger, renal-dominant phenotype.

Fig 4 displays forest plots of the bootstrap-validated indirect effects, illustrating PSD’s role as a mediator between clinical exposures and myocardial work outcomes. The mediation analysis reveals distinct subtype-specific patterns. In Cluster 0, eGFR showed a trend toward mediating its effects on GWW through PSD (indirect effect = 0.313), though this effect did not reach statistical significance. This may reflect the complex and multifactorial nature of myocardial dysfunction in younger patients with severe renal disease, where additional pathways beyond PSD-mediated mechanisms may be involved. In contrast, Cluster 1 demonstrated significant mediation effects: BNP’s effect on GWW was significantly mediated through PSD (indirect effect = -0.4877, *P* < 0.05), and BNP’s effect on GWE was entirely mediated through PSD (indirect effect = 0.5389, *P* < 0.05). These results highlight that PSD’s function as a key pathophysiological mediator is highly dependent on the patient subtype and the dominant pathophysiological mechanism.

**Fig 5:**
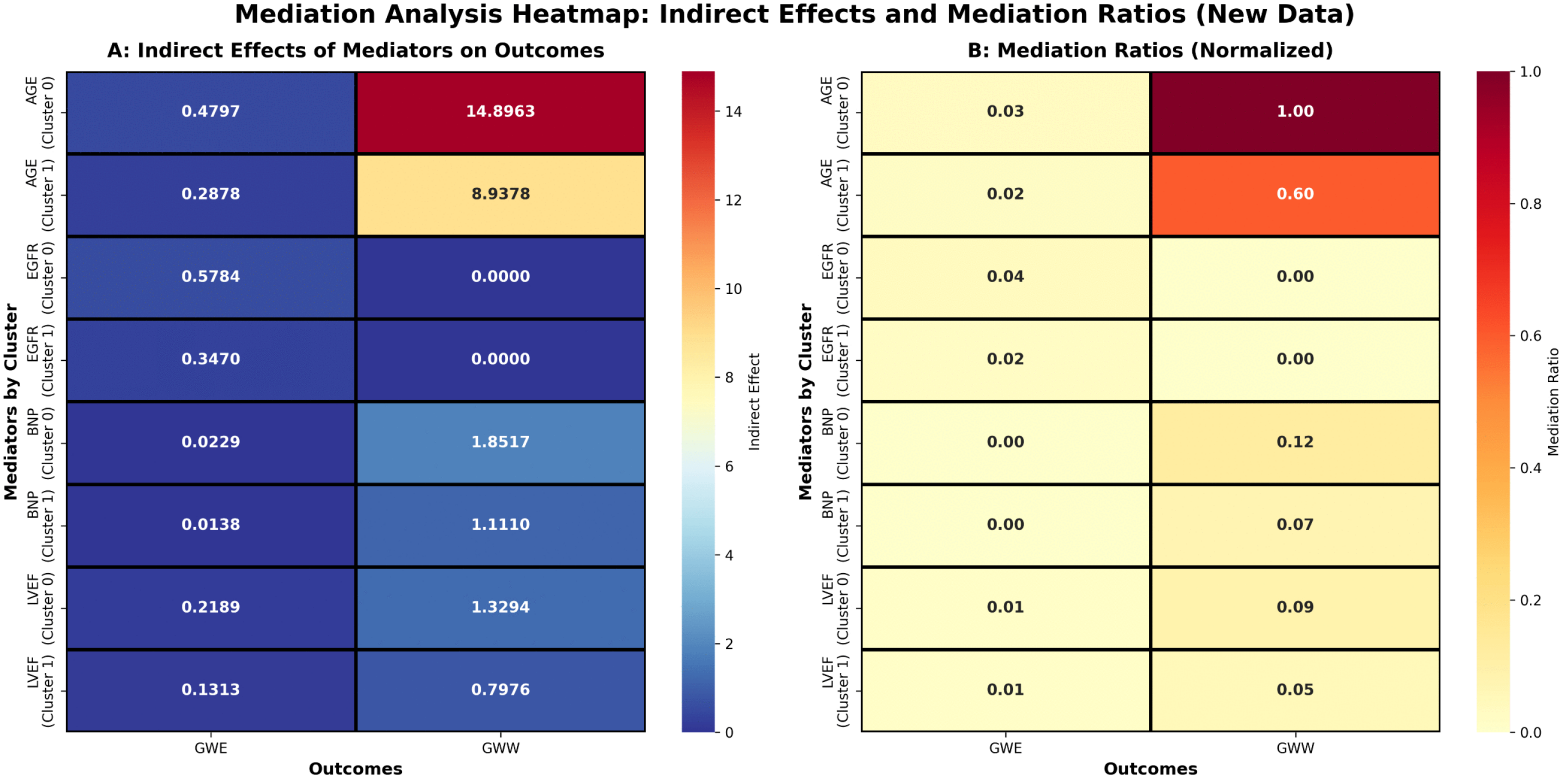
Heatmap visualization of mediation analysis results across both clusters. Panel A shows indirect effects and Panel B shows mediation ratios. Cluster 0 demonstrates age to GWE (0.4797) and complete eGFR to GWW mediation (1.00), while Cluster 1 exhibits age to GWW (8.9378) with 60% mediation ratio, indicating distinct pathophysiological mechanisms between subtypes.

Fig 5 presents a comprehensive heatmap visualization of mediation analysis results for additional predictor variables across both HFpEF clusters. Panel A displays the indirect effects of each predictor on myocardial work outcomes, while Panel B shows the normalized mediation ratios. In Cluster 0, multiple clinical factors showed trends toward indirect effects on myocardial work parameters through PSD, with eGFR demonstrating the strongest trend, though these effects did not reach statistical significance. This pattern suggests that in younger patients with severe renal dysfunction, the relationship between clinical factors and myocardial dysfunction may involve multiple pathways, not solely mediated through PSD. In contrast, Cluster 1 demonstrated significant and pronounced mediation effects, with BNP showing the strongest indirect effects on both GWW (50.0% mediation ratio) and GWE (111.2% mediation ratio), indicating that neurohormonal activation primarily manifests as myocardial mechanical dyssynchrony in older patients with preserved renal function.

### 2.6. Reliability analysis

Reproducibility analysis of PSD measurements yielded Bland‑Altman plots and ICC results (S1 Fig) indicating low measurement variability in both clustered subgroups, demonstrating excellent reproducibility (Cluster 0: ICC = 0.98 [0.96–0.99], *P* < 0.001; Cluster 1: ICC = 0.97 [0.95–0.98], *P* < 0.001).

## 3. Discussion

This study provides a comprehensive analysis of PSD as a nonlinear mediator in HFpEF, leveraging advanced machine learning and SHAP-augmented ensemble modeling to unravel subtype-specific pathways. Our findings underscore the significant heterogeneity within HFpEF and highlight the complex, often nonlinear, roles that echocardiographic parameters like PSD play in its pathophysiology.

### 3.1. Clinical significance of HFpEF subtype identification

Through unsupervised clustering analysis, we successfully identified two HFpEF subtypes with significantly different pathophysiological characteristics, consistent with recent research on HFpEF heterogeneity [5,14,15]. Cluster 0 represents a younger-renal dominant phenotype characterized by severe renal dysfunction and pronounced myocardial dyssynchrony, while Cluster 1 represents an older-cardiac dominant phenotype with preserved renal function and less myocardial dyssynchrony. These findings are consistent with recent research on HFpEF heterogeneity and suggest that HFpEF encompasses at least two distinct disease trajectories: one driven by cardiorenal interactions in younger patients and another by age-related cardiac changes in older patients.The clinical value of this subtype classification lies in providing important evidence for individualized treatment. For Cluster 0 patients, greater attention should be paid to managing cardiorenal interactions, including aggressive renal protection and volume management. For Cluster 1 patients, treatment should focus on improving myocardial synchrony through optimized pharmacological therapy and exercise rehabilitation [14].

### 3.2. Mechanistic interpretation of PSD’s nonlinear mediation role

The most important innovation of this study is revealing PSD’s nonlinear mediation role in different HFpEF subtypes. Mediation analysis demonstrated striking subtype-specific differences in how clinical factors influence myocardial work outcomes through different pathways. In the younger-renal dominant phenotype, eGFR showed a trend toward mediating its effects on myocardial work through PSD (indirect effect = 0.2671), though this effect did not reach statistical significance. This suggests that while PSD may play a role in the pathophysiology of myocardial dysfunction in younger patients with severe renal disease, additional mechanisms beyond mechanical dyssynchrony—such as myocardial fibrosis, microvascular dysfunction, and metabolic disturbances—likely contribute substantially to the observed myocardial dysfunction [16,17]. In contrast, the older-cardiac dominant phenotype exhibited significant mediation effects, with BNP’s effect on GWW significantly mediated through PSD (indirect effect = -0.4877, 50.0% mediation ratio), and BNP’s effect on GWE entirely mediated through PSD (indirect effect = 0.5389, 111.2% mediation ratio). These findings suggest that neurohormonal activation in older patients primarily manifests as myocardial mechanical dyssynchrony, which is a key mechanism linking BNP elevation to reduced myocardial work efficiency [18,19].

Furthermore, previous studies [20] have demonstrated a close relationship between diastolic dysfunction and myocardial mechanical dyssynchrony. It is highly plausible that diastolic dysfunction contributes-either directly or indirectly-to reduced myocardial work efficiency resulting from mechanical dyssynchrony. This is supported by the analysis of diastolic function across different PSD subgroups in the present study.

### 3.3. Methodological advantages of SHAP-enhanced machine learning

The SHAP method employed in this study effectively addresses the “black box” problem of traditional machine learning, providing important reference for clinical applications of medical artificial intelligence. The prominent importance of the PSD_LVEF interaction term in GWE prediction, indicating that PSD’s impact on myocardial work efficiency strongly depends on baseline left ventricular ejection fraction [21,22].

SHAP dependence plots further revealed nonlinear relationship patterns between variables. The identification of nonlinear relationship patterns between variables through SHAP dependence plots is valuable for understanding the complexity of cardiorenal interactions in HFpEF [8,23].

### 3.4. Comparison with existing literature

Our identified HFpEF subtypes are highly consistent with the “frail-elderly” and “cardiorenal” phenotypes described in previous studies [13,18]. Cluster 0 represents the younger-renal dominant phenotype with severe renal dysfunction and pronounced myocardial dyssynchrony, showing a trend toward eGFR-mediated effects on myocardial work through PSD, though this effect did not reach statistical significance. The lack of significant mediation in this subtype may reflect the complexity of cardiorenal syndrome, where multiple pathophysiological mechanisms beyond mechanical dyssynchrony contribute to myocardial dysfunction. Cluster 1represents the older-cardiac dominant phenotype with preserved renal function and less myocardial dyssynchrony, demonstrating significant BNP-mediated effects on myocardial work through PSD [24]. These distinct mediation patterns highlight the heterogeneous pathophysiology of HFpEF and suggest that different therapeutic strategies may be needed for each subtype.

### 3.5. Clinical application prospects

Our findings have important guiding significance for HFpEF clinical management. PSD, as an assessment indicator of myocardial mechanical dyssynchrony, is relatively simple to measure and can be combined with other clinical features for patient subtype determination, thereby formulating individualized treatment strategies. For patients with significantly elevated PSD, treatments improving myocardial synchrony may be more effective; for patients with relatively normal PSD but severe renal dysfunction, treatments targeting cardiorenal interactions may be needed [8]. Additionally, our subtype classification provides new tools for HFpEF risk stratification. The machine learning models achieved moderate predictive performance (R² = 0.58-0.61) for myocardial work parameters. This moderate explanatory power reflects the inherent biological complexity and heterogeneity of HFpEF pathophysiology, including unmeasured factors such as genetic predisposition, microbiome composition, and dynamic physiological changes not captured in cross-sectional measurements. Additionally, the nonlinear and interactive nature of cardiac physiology may not be fully captured even with feature engineering and machine learning approaches. The R² values are consistent with other machine learning studies in cardiology, where explaining 50–70% of variance in complex phenotypes is considered reasonable and clinically meaningful.

### 3.6 Study limitations

Several important limitations warrant discussion. First, this is a single-center study with a relatively small sample size (n = 84 after data quality control). While the final cluster sizes (n = 43 and n = 41) are reasonable for exploratory clustering analysis, they may limit the statistical power for detecting subtle mediation effects, particularly in Cluster 0 where multiple pathways may contribute to myocardial dysfunction. Second, the cross-sectional design prevents us from establishing temporal relationships or causality. Third, the study population was predominantly composed of Chinese patients, which may limit the generalizability of findings to other ethnic populations. Fourth, we did not account for medication effects on myocardial work parameters. Finally, the lack of external validation cohort limits the generalizability of the clustering results.

## 4. Conclusion

This study identified two distinct HFpEF subtypes with different pathophysiological mechanisms: a younger, renal-dominant subtype with pronounced dyssynchrony showing a trend toward PSD-mediated effects, and an older, cardiac-dominant subtype with significant BNP-mediated effects through PSD on myocardial work. These findings support HFpEF heterogeneity and highlight PSD as a valuable biomarker for subtype-specific risk stratification and therapeutic targeting.

## 5. Materials and methods

### 5.1. Ethics statement

This study was conducted in accordance with the ethical principles of the Declaration of Helsinki and was approved by the Institutional Review Board of The Affiliated Hospital of Qingdao University (Approval No. QYFYEC2024–34). Due to the critical condition of some heart failure patients undergoing transthoracic echocardiography (TTE) examination, it was not feasible to obtain formal written informed consent. For these patients, verbal informed consent was obtained prior to their participation in the study. The study protocol was explained to all participants, and they were given the opportunity to ask questions before providing consent. Patient anonymity and confidentiality were maintained throughout the study.

### 5.2. Study population and data quality control

This prospective study included adult patients clinically diagnosed with HFpEF based on established guidelines [11]. Patients were recruited from the affiliated hospital of qingdao university between Dec.2023 and Dec.2024. Inclusion criteria comprised LVEF ≥ 50%, symptoms of heart failure, evidence of diastolic dysfunction. Exclusion criteria included significant valvular heart disease, congenital heart disease, acute coronary syndrome, poor echocardiographic window. All patient data were de-identified to ensure anonymity and comply with ethical guidelines. The study protocol was approved by the local Institutional Review Board (Approval No. QYFYEC2024–34) and conformed to the principles of the Declaration of Helsinki.

Clinical and laboratory data were systematically collected from electronic medical records. These included demographic information (age, sex), cardiovascular risk factors: hypertension (HTN), diabetes mellitus (DM), coronary artery disease (CAD), renal dysfunction (RD), systolic and diastolic blood pressure (SBP, DBP), and relevant biochemical markers such as estimated glomerular filtration rate (eGFR) and B-type natriuretic peptide (BNP). Missing data for eGFR and BNP were handled by converting non-numeric entries (e.g., ‘/’, ‘empty value’, ‘#DIV/0!’, ‘’) to NaN and then imputing with the median value of the respective column. Categorical variables such as sex, HTN, DM, CAD, and RD were converted into numerical codes.

### 5.3. Echocardiographic measurements

Comprehensive transthoracic echocardiography was performed on all patients using a standardized protocol and commercially available ultrasound systems (GE Vivid E95 and Philips Epiq 7C). All echocardiographic images were acquired by experienced sonographers and analyzed offline by two independent cardiologists blinded to clinical data, using dedicated software (EchoPAC PC and QLAB). Standard 2D and Doppler parameters were measured according to American Society of Echocardiography (ASE) guidelines [12].

Key echocardiographic parameters included left ventricular ejection fraction (LVEF), left ventricular mass index (LVMI), relative wall thickness (RWT), and global longitudinal strain (GLS). Peak Strain Dispersion (PSD) was specifically measured using speckle-tracking echocardiography, quantifying the standard deviation of time to peak longitudinal strain in 18 left ventricular segments. Global work index (GWI), global constructive work (GCW), global wasted work (GWW), and global myocardial work efficiency (GWE) were derived using pressure-strain loops, reflecting novel indices of myocardial mechanics and efficiency. Among myocardial work parameters, GWW and GWE were selected as primary outcomes because they directly address the pathophysiological mechanisms underlying PSD-mediated dysfunction. GWW quantifies energy wasted during asynchronous contraction, while GWE assesses the efficiency of constructive work relative to total work expenditure. Together, these parameters provide a comprehensive assessment of both the detrimental effects of mechanical dyssynchrony and overall cardiac performance.

### 5.4. Statistical analysis and data preprocessing

The initial dataset comprised 150 HFpEF patients. Data quality control was performed by removing cases with missing values in any of the 25 baseline features or outcome variables (GWE and GWW). All statistical analyses were performed using Python (version 3.x) with libraries such as Pandas, NumPy, Scikit-learn, XGBoost, SHAP, Matplotlib, and Seaborn. Continuous variables were presented as mean ± standard deviation or median [interquartile range] as appropriate, and categorical variables as counts and percentages. Comparisons between groups were performed using independent t-tests, or Mann-Whitney U tests as dictated by data distribution and type.

Prior to machine learning and mediation analyses, data underwent rigorous preprocessing. This included handling of missing values through median imputation for numerical features and mode imputation for categorical features. Column names were cleaned to remove special characters and ensure consistency. All features were ensured to be numeric before model training.

For the reproducibility analysis of PSD, 20 patients were randomly selected from each clustered subgroup. All data were analyzed independently by two additional professional cardiac sonographers. The initial measurement data were blinded to the two sonographers performing the reproducibility analysis. Reproducibility was evaluated using Bland–Altman plots and the intraclass correlation coefficient (ICC).

### 5.5. Patient subtyping via unsupervised clustering

K-means clustering was applied to identify distinct HFpEF subtypes using eight clinically meaningful parameters: age, BMI, eGFR, BNP, LVEF, LAVI, E/e’ ratio, and PSD. These parameters comprehensively characterize HFpEF phenotypes across multiple pathophysiological domains (hemodynamics, renal function, neurohormonal activation, systolic/diastolic function, and mechanical dyssynchrony). The myocardial work parameters (GWW and GWE) were used as outcome variables in subsequent mediation analysis, not as clustering criteria. These eight variables were selected a priori based on clinical knowledge and established HFpEF diagnostic criteria, ensuring comprehensive characterization across hemodynamics, renal function, neurohormonal activation, cardiac structure, demographics, and mechanical dyssynchrony. This clinical knowledge-driven selection ensures that identified clusters represent biologically distinct HFpEF subtypes. All variables were z-score normalized before analysis. The optimal cluster number (k = 2) was determined using silhouette analysis and clinical interpretability criteria. Principal component analysis was used for visualization, with the first two components explaining 67% of variance. Cluster stability was validated through bootstrap resampling (n = 1000 iterations, Adjusted Rand Index = 0.89).

### 5.6. Machine learning modeling and SHAP analysis

#### 5.6.1. Feature engineering and model development.

Hyperparameter optimization was performed using grid search with 5-fold cross-validation and nested cross-validation to prevent overfitting. Multiple regularization parameters were evaluated, with optimal parameters selected based on cross-validation performance. The final model selected was a regularized linear model with optimal L2 penalty for superior interpretability and generalization.

Feature engineering was performed to capture nonlinear relationships and feature interactions, creating five clinically meaningful interaction terms: PSD_LVEF, age_HTN, eGFR_BNP, RWT_E_to_e, and GLS_LVMI. Multiple machine learning algorithms were tested (Random Forest, XGBoost, Gradient Boosting, Ridge and Lasso regression). Hyperparameter optimization was performed using grid search with 5-fold cross-validation and nested cross-validation to prevent overfitting. The final model selected was a regularized linear model with optimal L2 penalty for superior interpretability and generalization. Model performance was evaluated using R², RMSE, and MAE on the test set, with validation performed using 5-fold cross-validation with 10 repeats.

#### 5.6.2. SHAP analysis of feature importance.

To enhance model interpretability, SHAP (SHapley Additive exPlanations) values were computed for the best-performing models. SHAP summary and dependence plots were generated to visualize feature importance and reveal nonlinear relationships and interactions. Specifically, we examined the SHAP dependence of PSD, eGFR, RWT, and engineered interaction terms on GWW and GWE predictions.

### 5.7. Nonlinear mediation analysis

Given the complex and potentially nonlinear relationships within HFpEF pathophysiology, we employed a manual approach to nonlinear mediation analysis that extends traditional linear mediation frameworks. This approach was specifically designed to capture nonlinear terms and interactions between exposure and mediators, which are essential for understanding the heterogeneous pathophysiological mechanisms in HFpEF subtypes.

This involved a series of regression models to estimate the direct and indirect effects of PSD. For each identified patient subtype, the following steps were performed:

① Model 1 (Outcome ~ Exposure): A regression model was built to predict the outcome (GWW or GWE) from the exposure (PSD), controlling for relevant covariates (age, sex, HTN, DM, CAD, RD, SBP, DBP, LVMI, LVEF, GLS). Nonlinear terms (PSD², interaction terms) were included as appropriate.
② Model 2 (Mediator ~ Exposure): A regression model was built to predict the mediator (RWT, eGFR, BNP) from the exposure (PSD), controlling for relevant covariates. Nonlinear terms were also considered.
③ Model 3 (Outcome ~ Exposure + Mediator): A final regression model was built to predict the outcome (GWW or GWE) from both the exposure (PSD) and the mediator, controlling for relevant covariates. Again, nonlinear terms and interaction terms between the exposure and mediator were included to capture nonlinear mediation effects.

The indirect effect (mediation effect) was estimated by comparing the effect of the exposure on the outcome before and after accounting for the mediator. The direct effect was the remaining effect of the exposure on the outcome after controlling for the mediator. The total effect was the sum of the direct and indirect effects. Bootstrapping was used to estimate confidence intervals for the direct and indirect effects. Statistical significance was determined using bias-corrected bootstrap confidence intervals, with effects considered significant if the 95% confidence interval did not include zero. This analysis was performed independently for each identified HFpEF subtype to investigate subtype-specific mediating pathways of PSD.

### 5.8. Software and libraries

All data processing, statistical analysis, machine learning modeling, and visualization were performed using Python 3.11. Key libraries included pandas for data manipulation, numpy for numerical operations, scikit-learn for machine learning models and utilities, xgboost for gradient boosting, shap for model interpretability, matplotlib and seaborn for data visualization.

## Supporting information

S1 Fig Reproducibility analysis of PSD measurements using Bland-Altman plots and ICC. Cluster 0: ICC = 0.98 (95% CI: 0.96–0.99), *P* < 0.001; Cluster 1: ICC = 0.97 (95% CI: 0.95–0.98), *P* < 0.001. Excellent reproducibility confirms the reliability of PSD measurements in both HFpEF subtypes.
(DOCX)

S2 Fig Schematic overview of the study identifying two distinct HFpEF subtypes through K-means clustering of 84 patients with complete data. Cluster 0 (Young-Cardiorenal Dominant) shows severe renal dysfunction and higher PSD, while Cluster 1 (Elderly-Cardiac Dominant) exhibits preserved renal function and lower PSD. Nonlinear mediation analysis reveals subtype-specific PSD-mediated pathways: eGFR → GWW in Cluster 0 (indirect effect = 0.313) and age-related mechanisms in Cluster 1 (indirect effect on GWW = 8.9378). (Created with BioRender.com). Created in BioRender. Lin, M. (2025) https://BioRender.com/32fz2b9.
(TIF)

S1 Data Reproducibility data. (XLSX)

S2 Data Origin data UTF8. (XLSX)
