# Supplementary material for: Peak strain dispersion as a nonlinear mediator in HFpEF: Unraveling subtype-specific pathways via SHAP-augmented ensemble modeling
Source: PLoS Comput Biol. 2026 Jan 14;22(1):e1013891. doi: 10.1371/journal.pcbi.1013891 (PMC12829950; doi:10.1371/journal.pcbi.1013891)
Supplement: S1 Fig — Cluster 0: ICC = 0.98 (95% CI: 0.96–0.99), P < 0.001; Cluster 1: ICC = 0.97 (95% CI: 0.95–0.98), P < 0.001. Excellent reproducibility confirms the reliability of PSD measurements in both HFpEF subtypes. (DOCX) [file pcbi.1013891.s001.docx]

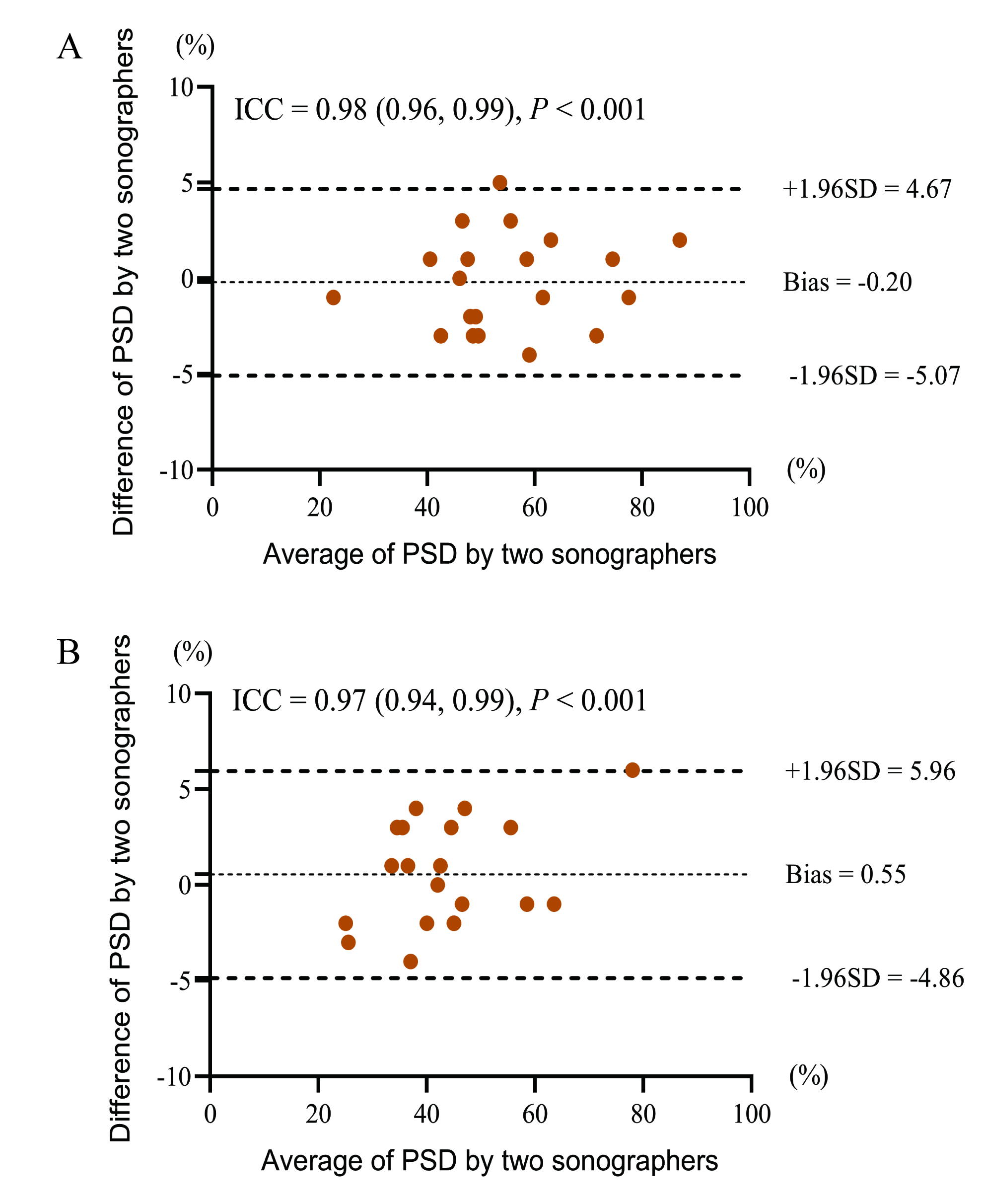


Supplemental Fig.1 Reproducibility analysis of PSD measurements by two sonographers

ICC, intra class coefficient; PSD, peak strain dispersion
